# Synthesis of “Yellow Cobblestones” of Sofia Using Asbestos Waste

**DOI:** 10.3390/ma19204330

**Published:** 2026-10-15

**Authors:** Alexander Karamanov, Vladislav Kostov-Kytin, Emilia Karamanova

**Affiliations:** 1 Institute for Physical Chemistry, Bulgarian Academy of Sciences, IPC-BAS, Bl. 11, Acad. G. Bonchev Str., 1113 Sofia, Bulgaria; ekarama@ipc.bas.bg; 2 Institute of Mineralogy and Crystallography, Bulgarian Academy of Sciences, Acad. G. Bonchev Str., Bl. 107, 1113 Sofia, Bulgaria; vkytin@abv.bg

**Keywords:** ceramics, marl, asbestos, sintering, cultural heritage

## Abstract

**Highlights:**

- A material similar to Sofia’s historic “yellow cobblestones” has been synthesized.
- The structure and phase composition of the new ceramic are comparable to those of the original.
- Bulgarian marl was used as the main raw material.
- The only additive used was asbestos waste rich in MgO.

**Abstract:**

The “yellow cobblestones” of Sofia are considered one of the city’s symbols. This high-quality clinker was developed in Budapest in the late 19th century by using a local marl. Since this paving was declared part of Sofia’s cultural heritage, studies to synthesize similar materials were carried out. Unfortunately, the Bulgarian clays used are characterized by a lower MgO amount, which results in a very narrow sintering range. However, an increase in MgO in the parent batch, bringing the composition closer to that of the original, has a positive effect on sintering, phase composition and structure. This work discusses the possibility of obtaining similar clinker by adding asbestos waste containing a high percentage of MgO to a selected Bulgarian marl. The process involves pre-calcination of part of the marl together with the asbestos waste, followed by mixing the resulting grog with the remaining clay, homogenization, pressing and final firing. Studies with Hot Stage Microscopy and optical dilatometry demonstrate that the asbestos addition has a positive effect on the widening of the sintering interval, while XRD, SEM and pycnometric results elucidate that the samples obtained in this way are characterized by a phase composition and structure comparable to those of the original material.

## 1. Introduction

**Figure 1:**
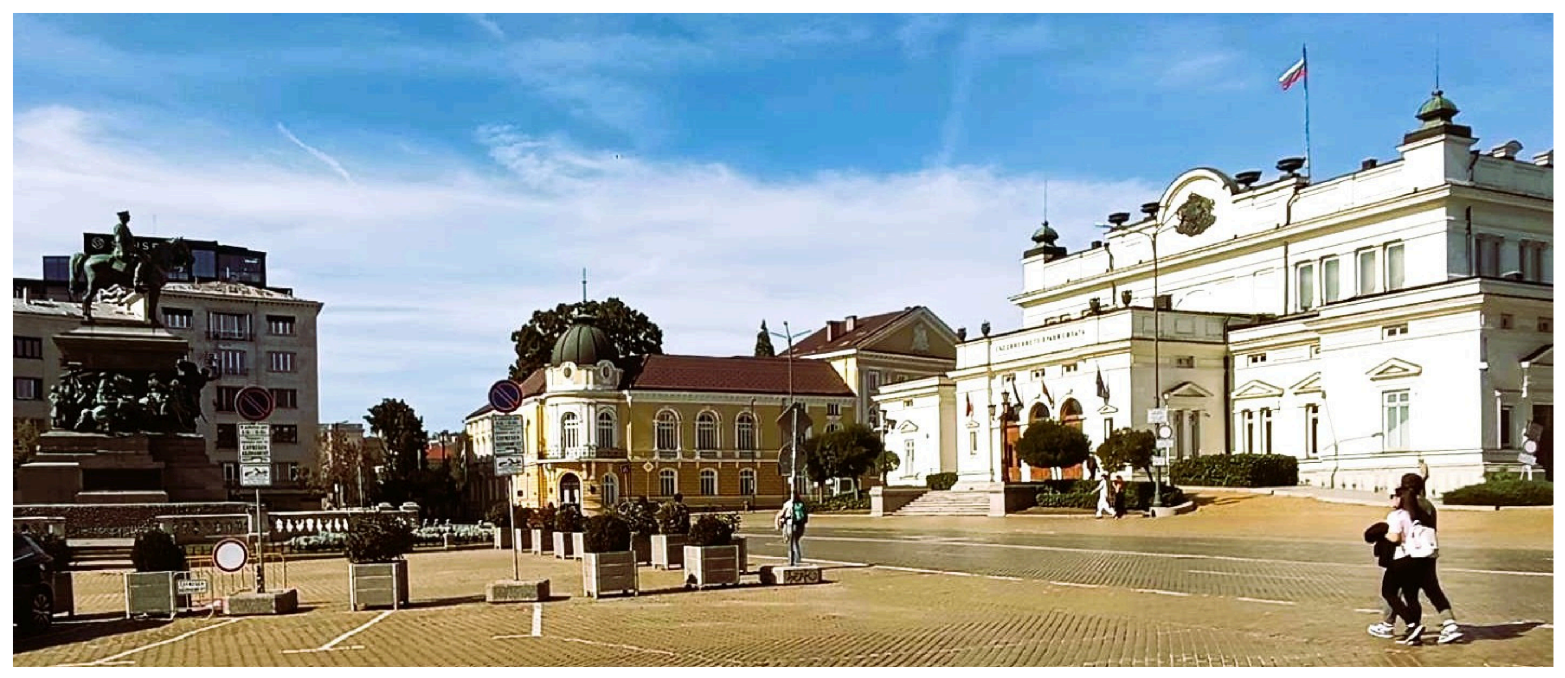
Central part of Sofia.

The yellow cobblestones in Sofia’s historic center are among the most recognizable symbols of the Bulgarian capital and, even today, remain an integral part of its cultural and urban identity. Laid between 1907 and 1909, this paving reflected the ambition of the young Bulgarian state to create a modern European capital. It covered around 60 decares of Sofia’s streets and squares, creating a unique architectural landscape. Today, this area is half what it once was, and the yellow cobblestones have been declared part of Sofia’s cultural heritage. A photo of the center of Sofia, covered with the yellow cobblestones, is shown in Figure 1.

In practical terms, this material is a very well-sintered clinker, imported from Budapest. This luxury pavement, named “Keramit”, was developed in the late 19th century by using local marl with a specific composition. The brick factory that produced this clinker was located in the region of Kőbánya (which means “Quarry”), which today is one of the largest districts of modern Budapest [1]. After the depletion of the pits and the expansion of Budapest in these areas, “Keramit” production was interrupted. Brick production in Budapest definitively ended in 1972 and the open pits became a heterogeneous municipal landfill and metro excavation site [2].

Our preliminary studies have shown that the original Keramit is characterized by near-zero water absorption and about 10 vol % closed porosity, coupled with elevated crystallinity and a fine crystalline structure based on pyroxene and anorthite solid solutions [3]. This combination explains its extraordinary mechanical properties and its ability to survive for more than a century under demanding urban conditions. It is curious that the sintering temperature of this marl ceramic is below those of modern clinkers, which are characterized by a lower degree of sintering and inferior mechanical characteristics. However, the production of this interesting material involves some technological problems which are not typical for the usual triaxial ceramics (i.e., those based on clay, quartz and feldspar). The main disadvantage, which is also typical for many well-sintered marl ceramics, is the relatively narrow sintering interval. The main reason for this is that due to the high % of CaO, an elevated amount of liquid phase with a relatively low viscosity is rapidly formed once the eutectic temperature is reached.

In traditional brick manufacturing, where the working temperatures are lower, this particularity is not so important. In this case it is risky if the marl clay contains coarser limestone inclusions, which, during firing, might turn into lumps of quicklime (CaO). Then these inclusions would absorb moisture and turn into calcium hydroxide, whose volume increases by nearly 3 times. For this reason, the raw marl must be thoroughly ground, and the heat treatment applied must ensure the complete incorporation of CaO into the newly formed aluminum silicate phases.

Despite these manufacturing challenges, many factories produce marl bricks for different compelling economic, practical or architectural reasons. As brickworks are usually built near clay pits, if the local mine yields marl clay, the production processes will be adapted to make use of it. Furthermore, yellow and beige marl bricks reflect more natural light than dark red bricks, giving contemporary urban architecture a softer and more luxurious appearance. The lighter color of the marl product is attributed to Fe^3+^ into the crystal lattices of calcium-bearing silicate minerals (e.g., plagioclases and pyroxenes) rather than occurring as discrete hematite phases. Consequently, the characteristic red coloration associated with hematite is significantly reduced [4,5,6].

A significant part of brick production in Northern Bulgaria is also based on marl clay. This was the reason that, in another of our studies, some of these clays were characterized and compared with the data reported for the marls near Budapest [7]. The results show that the utilized Bulgarian deposits contain about 50–60% clay minerals (mainly illite and chlorite), 15–20% calcite and 15–20% quartz, while in the Hungarian raw materials the quartz amounts werewas a little lower, some dolomite was present together with the calcite and part of the clay consisted of smectite.

These differences are not particularly significant for the traditional brick production, but they are crucial for the firing of a high-quality clinker. In fact, the absence of dolomite and the associated lower MgO content in the Bulgarian marls poses a serious problem due to the narrowing of the sintering range. This was the reason why the same study also identified suitable Bulgarian raw materials rich in MgO (such as dolomite, talc, magnesium carbonate, etc.) as possible additives to marl [7].

However, this paper investigates and demonstrates another interesting possibility relating to the use of asbestos waste as an additive.

Asbestos is a group of naturally occurring silicate minerals made of flexible, heat-resistant fibers which, thanks to their exceptional resistance to heat, fire, chemicals and electricity, have been used for hundreds of years. In recent decades, however, the use of asbestos has been restricted or banned in many countries. A comprehensive ban at the EU level was introduced by Commission Directive 1999/77/EC of 26 July 1999, which prohibited the placing on the market and the use of asbestos and products containing asbestos; member states were required to transpose the provisions of the Directive into their national legislation by 1 January 2005.

Whilst other minerals or industrial wastes are classified as hazardous due to their chemical composition and the high solubility of heavy metals, asbestos is toxic because of its structure, which consists of thousands of tiny, fibrous crystals. When inhaled into the lungs, these cause carcinogenic effects due to the constant irritation of the tissues.

The traditional methods for the disposal of wastes containing asbestos are based primarily on controlled landfill, which however does not eliminate long-term risks and requires specialized storage conditions. Growing pressure on landfill capacity, rising landfill costs and the principles of sustainable resource management have stimulated interest in alternative processing and recycling strategies capable of transforming hazardous asbestos-containing waste into environmentally safe products with benefits.

Over the past decade, it has also been demonstrated that thermally decomposed wastes containing asbestos can be used as a suitable raw material for the manufacture of various ceramic products [8,9,10,11]. At temperatures of 600–800 °C, asbestos minerals lose their chemically bound water and transform into a metastable phase with high reactivity; upon reaching 1000–1050 °C, recrystallization begins and non-toxic crystals are formed (usually a mixture of enstatite and forsterite). At higher temperatures, the vitrification technique is also available [12]; however, it is considerably more expensive.

When thermally treated waste materials containing very high amounts of asbestos are used, this significantly increases the magnesium oxide content in the ceramic, which in many cases is decisive for its structure and properties. This is exactly the case discussed in the present study.

## 2. Materials and Methods

### 2.1. Raw Materials

The marl used is from a quarry near the town of Popovo, North-Eastern Bulgaria, where bricks and roof tiles have been produced since the early 20th century. Thanks to the composition of this local marl, the fired products take on their characteristic yellowish-white color. As many of the houses and roofs in the town were built using these local ceramic products, nearly a century ago Popovo became known by the nickname “The White Town”.

Cords from an asbestos rope, which had been applied for insulation at high temperatures, were used as a MgO additive. Asbestos refractory ropes are positioned at the absolute top tier (Class 1/High Risk) of the global asbestos hazard hierarchy [13]. As a result, their landfilling is problematic and storage is expensive; the price is about 250 euro/ton or higher.

Initially, the asbestos waste was heat-treated with part of the marl to obtain a grog, which was then mixed with the remaining part of the marl to form the final ceramic batch.

### 2.2. Experimental Procedure

The chemical compositions of the raw materials, the original clinker and the new ceramic were evaluated by XRF analysis (Zetium Spectrometer—Malvern Panalytical, Almelo, The Netherlands).

The XRD patterns of the powdered samples were recorded on an Empyrean Powder X-ray diffractometer (Malvern Panalytical, Almelo, The Netherlands) using Cu radiation (λ = 1.5406 Å) at 40 kV and 30 mA and a PIXcel3D detector. The phase evaluation was carried out using the HighScore Plus software (version 5.2, Malvern Panalytical, The Netherlands).

In order to increase MgO in the ceramic, 2 g of asbestos cords was added to 10 mL water and 18 g marl. Then, this wet mix was milled for 30 min in a closed agate jar and transferred into a corundum crucible with a cap. All these procedures were carried out in a laboratory fume cupboard. Then the batch was heat-treated at slow heating rate of 2 °C/min up to 1000 °C and held for 1 h. The grog obtained in this way, labeled G*9/1, was milled to below 125 microns.

The sintering trends of G*9/1 and the original marl ***M*** were compared by Hot Stage Microscopy (HSM) tests at 10 °C min^−1^ (ESS HSM-1400 Expert System, Modena, Italy).

The parent batch of the new ceramics was prepared by mixing 70% of the milled grog with 30% of the parent marl, moistening with 7% water, homogenization and uniaxial pressing at 50 MPa. The obtained “green” samples with a size of 50 × 5× (7–8) mm were subjected to a preliminary thermal treatment in an electric furnace up to 1100 °C at 3 °C/min. Due to decarbonization of the marl part, this heat treatment was accompanied by a total shrinkage of about 1%. Then the optimal firing schedule was determined using an optical dilatometer and 4 h holding at different temperatures in the range of 1140–1180 °C (ESS OHD-1400 Expert System, Modena, Italy). A heating rate of 10 °C/min was used up to 1100 °C, and was then reduced to 5 °C/min.

The apparent, ***ρ_a_***, skeleton, ***ρ****_s_*, and absolute, ***ρ_as_***, densities of the new ceramic, labeled ***C9/1***, and the original clinker ***K*** and their water absorption, ***WA***, were determined. ***ρ_a_*** was estimated with a precise micrometer and balance, while ***ρ_s_*** and ***ρ_as_*** were estimated with a gas pycnometer (AccyPy1330, Micromeritic, Norcross, GA, USA) before and after crushing and milling the samples below 26 μm, respectively. ***WA*** was measured after 3 h boiling in distillate water with a conductivity of less than 5 µS·cm^−1^. Then, the results were used to evaluate closed, ***P_C_***, and open, ***P_O_***, porosities:

(1) PC=100×(ρas−ρs)ρas

(2) $$P_{O}=WA\times \rho_{a}$$

Finally, the structure of the ceramic was studied using scanning electron microscopy (JEOL JSM 6390, Tokyo, Japan) after polishing the samples and performing Au metallization. The chemical compositions of the formed crystal phases were estimated by EDS analysis (INCA Oxford, High Wycombe, Buckinghamshire, United Kingdom).

## 3. Results and Discussion

The results from XRF chemical analysis of “Keramit”—***K***, marl—***M*** and asbestos waste—***A*** are given in Table 1. The slightly higher SiO_2_ and the slightly lower Al_2_O_3_ percentages in the original ceramic can probably be explained by the presence of smectite in the Hungarian marls and of illite in the Bulgarian ones [7,14]. Another, more significant difference is the twice-as-high MgO content in the “Keramit” due to the presence of dolomite in the marl from the quarries in Budapest. However, this can easily be compensated for by adding a certain quantity of asbestos waste to the Bulgarian raw material. Furthermore, such an adjustment would not reduce the total quantity of SiO_2_, which can also be regarded as a positive effect. It can be assumed that no other additives are required for this batch.

**Figure 2:**
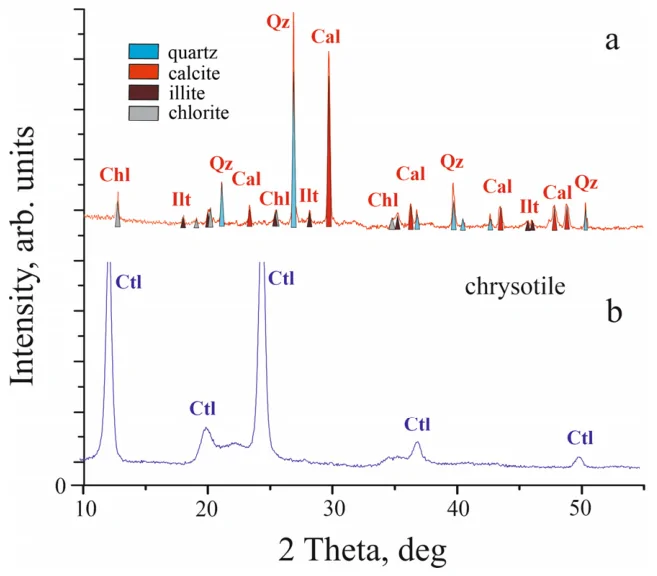
XRD patterns of initial marl (**a**) and asbestos rope (**b**).

The XRD results for the parent raw materials are shown in Figure 2. The marl contains mainly limestone, quartz, illite and chlorite, while the asbestos cords are made essentially of chrysotile—Mg_3_(Si_2_O_5_)(OH)_4_. The measured losses on ignition at 1000 °C (LOIs) are 19.3% for ***M*** and 16.2% for ***A***. These values are typical for similar samples and are consistent with both the chemical and phase analyses.

**Figure 3:**
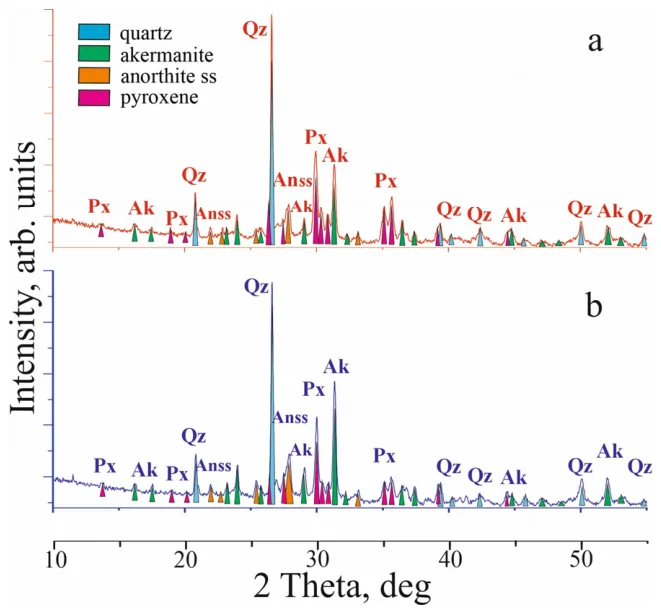
XRD patterns of grog G*9/1 (**a**) and initial marl ***M*** (**b**) after 1 h holding at 1000 °C.

The XRD pattern of the grog G*9/1 obtained after 1 h holding at 1000 °C is shown in Figure 3 together with that of marl ***M***, which was heat-treated using the same regime. The result for sample G/*9/1 shows no traces of chrysotile, which confirms the successful thermal inertization. Residual quartz as well as newly formed crystalline phases belonging to the pyroxene group and to representatives of the melilite (Gehlenite–Åkermanite Series—Ca_2_Al_2_SiO_7_—Ca_2_MgSi_2_O_7_) and anorthite solid solutions are observed for this sample. The pattern of ***M*** is somewhat similar, but the amounts of melilite and anorthite are higher, while that of pyroxene is lower.

These results are reasonable because the melilite solid solutions are formed as a precursor phase due to the reaction between the products of decarbonization and dehydroxylation in the marls [15,16,17,18,19]. With the increasing of temperature, the melilite reacts with the remaining free silica and alumina to form anorthite and pyroxenes. It is also evident that the higher MgO content in sample G*9/1 leads to an increase in the ratio of pyroxenes to anorthite. As the rate of quartz dissolution at this temperature is low, its quantity remains significant. These reactions can be regarded as an example of the Ostwald’s rule [20] for the initial formation of kinetically more favorable non-equilibrium melilite solid solutions. Subsequently, these metastable phases decompose to form the equilibrium pyroxenes and anorthite.

**Figure 4:**
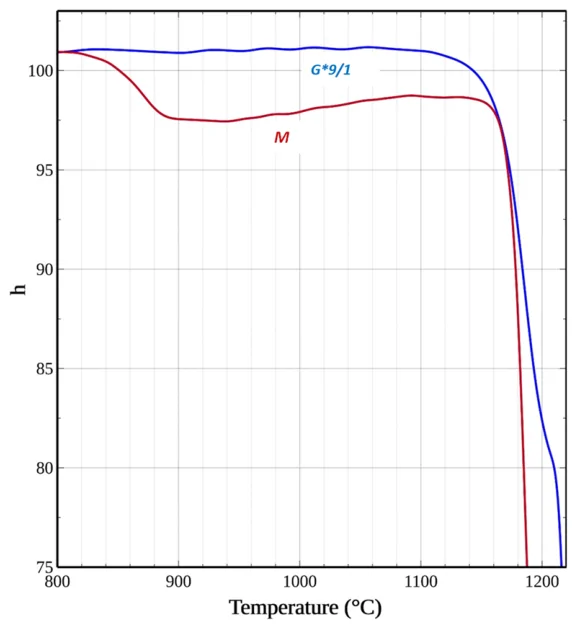
HSM results for the height variation (%) of G*9/1 and initial marl ***M***.

**Figure 5:**
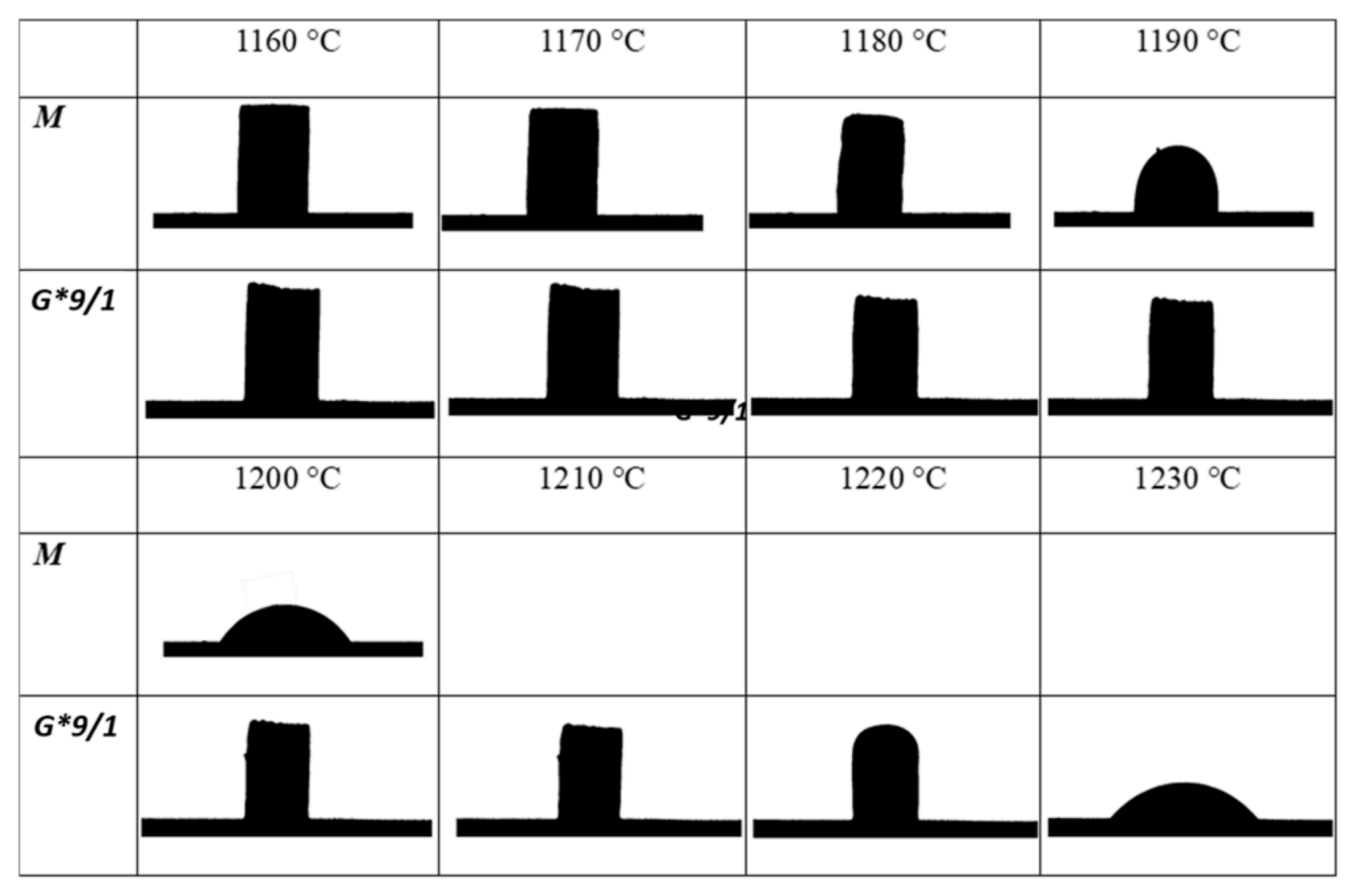
Silhouettes of G*9/1 and ***M*** at different temperatures.

In order to investigate the effect of asbestos addition on the sintering, heating stage microscope (HSM) tests were carried out. The shrinkage curves of samples G*9/1 and ***M*** are plotted in Figure 4, whilst Figure 5 shows the silhouettes of the two samples at different temperatures. The temperatures, corresponding to the “fixed” points according to HSM tests, and the corresponding apparent viscosities are summarized in Table 2 [21].

The results elucidate that in the temperature interval of decarbonization some shrinkage is observed in the parent marl, which obviously is missing in the previously heat-treated grog. After that, the sintering in G*9/1 starts earlier, while the softening, formation of the hemisphere and melting occur at significantly higher temperatures than in the marl sample. The “sintering interval” (i.e., the temperature difference between ***T_FS_*** and ***T_MS_***) is ~50 °C, after which the sample undergoes rapid melting. In the parent marl, it is practically difficult to identify the temperature of maximum shrinkage because it overlaps with the softening temperature. In this case the rapid eutectic melting is accompanied by a drop in apparent viscosity of approximately six orders of magnitude over a temperature interval of only 30 °C. Consequently, the sintering window of this composition is extremely narrow and the production of a well-sintered ceramic by using this raw material “as it is” is unrealistic.

The addition of asbestos waste in sample G*9/1 has a positive effect on the sintering interval, increasing it from approximately 10 to 50 °C. This result can be mainly related to the shift in the chemical composition from the eutectic region toward the pyroxene crystallization field [22], where the liquid–crystalline ratio is controlled by the lever rule and total melting occurs after reaching the corresponding liquidus temperature. A somewhat similar behavior has been reported and discussed in more detail in another of our publications [23]. In addition, some increase in the viscosity of the melt due to the partial replacement of CaO by MgO should also be taken into account [24,25].

However, the sintering interval remains relatively narrow, so the thermal treatment must be carefully controlled. To minimize the risk of deformation, firing at lower temperatures with a prolonged isothermal holding step is preferable.

The optimal regime for new ceramic ***C9/1***, obtained by mixing seven parts G*9/1 with three parts ***M***, was estimated with optical dilatometry. It can also be noted that the calcination of part of the marl improves the pressability of batch.

The results show that at 1140 °C the sintering was not completed, whereas at 1180 °C some deformation and swelling were observed. However, the samples fired at 1150, 1160, and 1170 °C for 4 h exhibited similar shrinkage values of 9.9 ± 0.2%. These results demonstrate good dimensional stability over a 20 °C firing interval (1150–1170 °C), which is acceptable for well-controlled production of paving clinker according to the dimensional requirements [26].

It can be also noted that no visual variations were observed in the color of these laboratory samples. Their appearance and color are comparable to those of the original “Keramit”, which, however, shows variations between yellow and brownish yellow (mainly due to partial reduction of Fe_2_O_3_ in the darker samples). In fact, some color variation is acceptable for clinker bricks.

**Figure 6:**
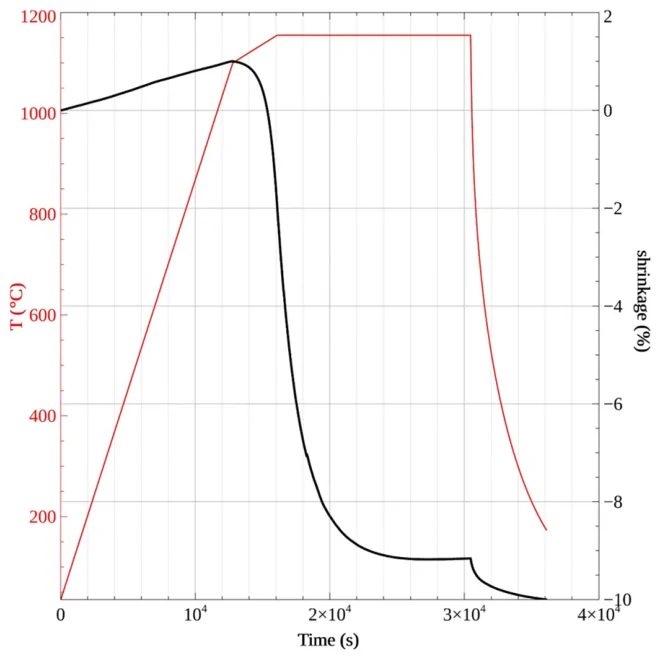
Dilatometry sintering curve of ***C9/1*** at 1160 °C.

The sintering curve of the optimal sample, fired at 1160 °C, is shown in Figure 6. The expansion at 1100 °C is about 1%, which corresponds to a realistic average value for the coefficient of linear thermal expansion of about 9.3 × 10^−6^/°C. Slow sintering then starts, and at the beginning of the step the shrinkage is about 0.5%. During the first hour of holding the densification proceeds intensively and the shrinkage reaches about 8%, whilst after two hours it reaches 9%. After that, no significant variations (sintering or overfiring) are observed. During cooling, the thermal contraction is similar to the expansion observed at heating.

This sample, labeled ***C9/1***, was used to compare the new ceramic with the original “Keramit”.

The chemical composition of the new sample is presented in Table 1 and shows that it is significantly closer to that of the original ceramic than the parent marl. However, a decrease in SiO_2_ content of about 3% can be noted, accompanied by a simultaneous small increase in the percentages of CaO, Al_2_O_3_, and MgO. It can be assumed that this change could slightly increase the final crystallinity, which, provided the same degree of sintering occurs, could enhance its mechanical properties.

The information on the densities and porosities of samples ***K*** and ***C9/1*** is summarized in Table 3. The results are very similar and show that both samples are very well sintered and are characterized by near-zero open porosity. However, the closed porosity of 10–11% is 2–4% higher than that of porcelain stoneware [27,28] or sanitary ware [29], where the open porosity is also negligible. This difference is probably due to some crystallization in the marl ceramics, which occurs during cooling and which is not typical for the traditional triaxial compositions. As the crystalline phase is denser than the corresponding melt, the phase formation leads to the creation of additional porosity [30,31,32]. This “extra” crystallization may explain the unusual combination of high crystallinity and an exceptional degree of sintering in “Keramit”, which results in its excellent mechanical properties. In fact, its characteristics (i.e., compressive strength of 290 ± 15 MPa and flexural strength of 45 ± 10 MPa) are significantly higher than the requirements for modern clinker pavers [3].

**Figure 7:**
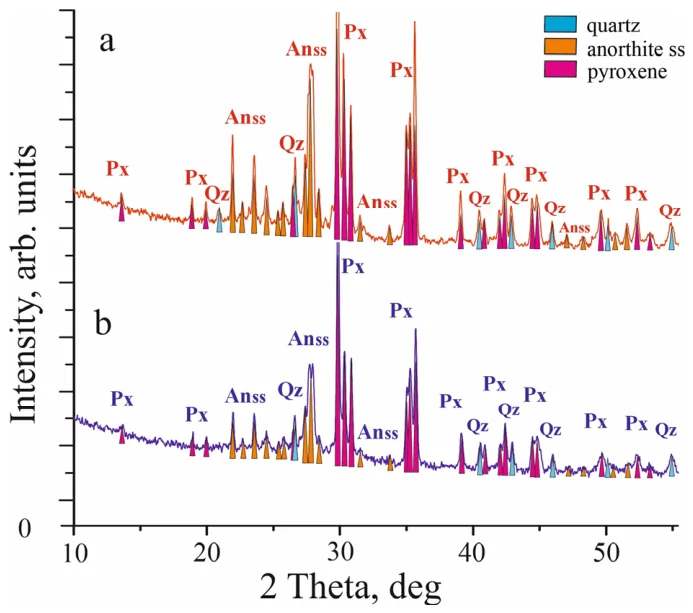
XRD patterns of original “Keramit” (**a**) and new ceramic ***C9/1*** (**b**).

The XRD analyses confirm the high crystallinity of both materials. The two patterns presented in Figure 7 are very similar and show an amorphous halo of low intensities and comparable ratios between pyroxene and anorthite phases. The main part of the quartz is dissolved, resulting in significantly reduced intensity of its peaks (see Figure 2 and Figure 3). In our previous study, using the Rietveld method, it was estimated that the crystallinity of the original clinker exceeds 65% [33].

The very good degree of sintering and the high crystallinity were also confirmed by SEM observations.

**Figure 8:**
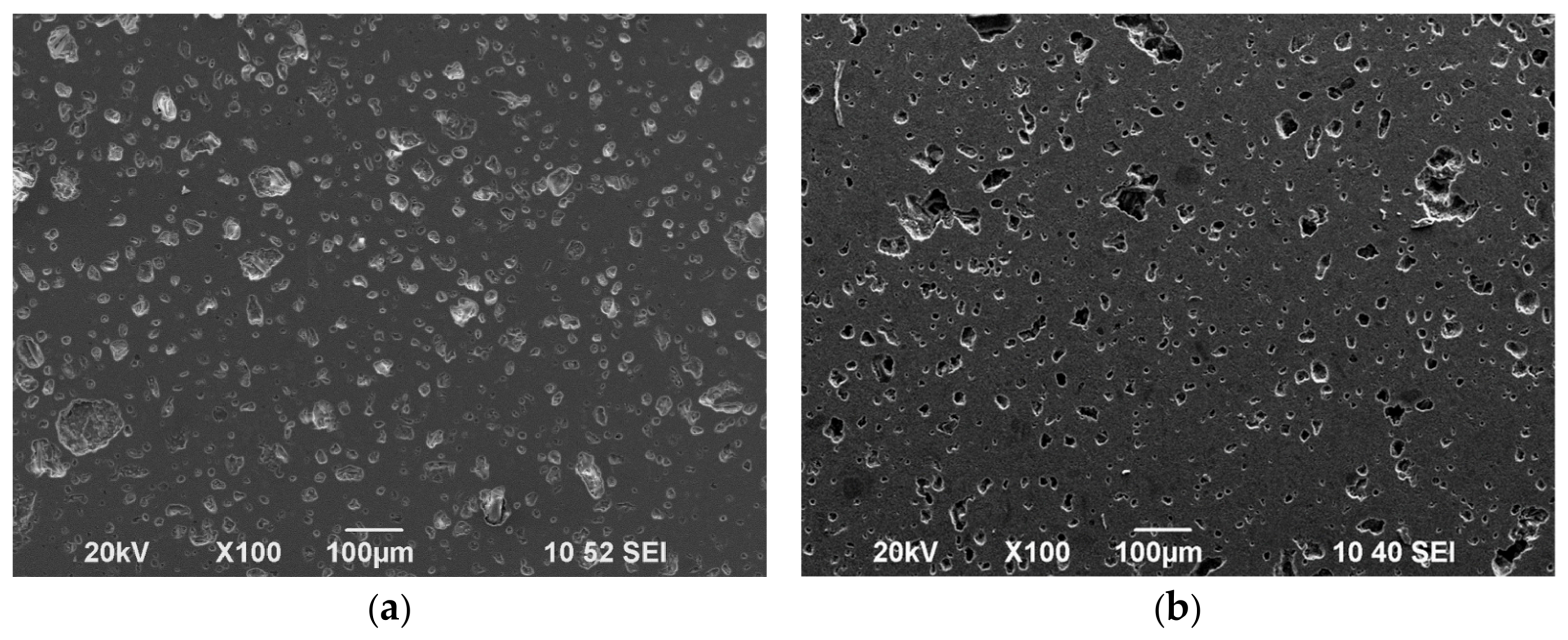
Polished fracture surfaces of original “Keramit” (**a**) and new ceramic ***C9/1*** (**b**).

**Figure 9:**
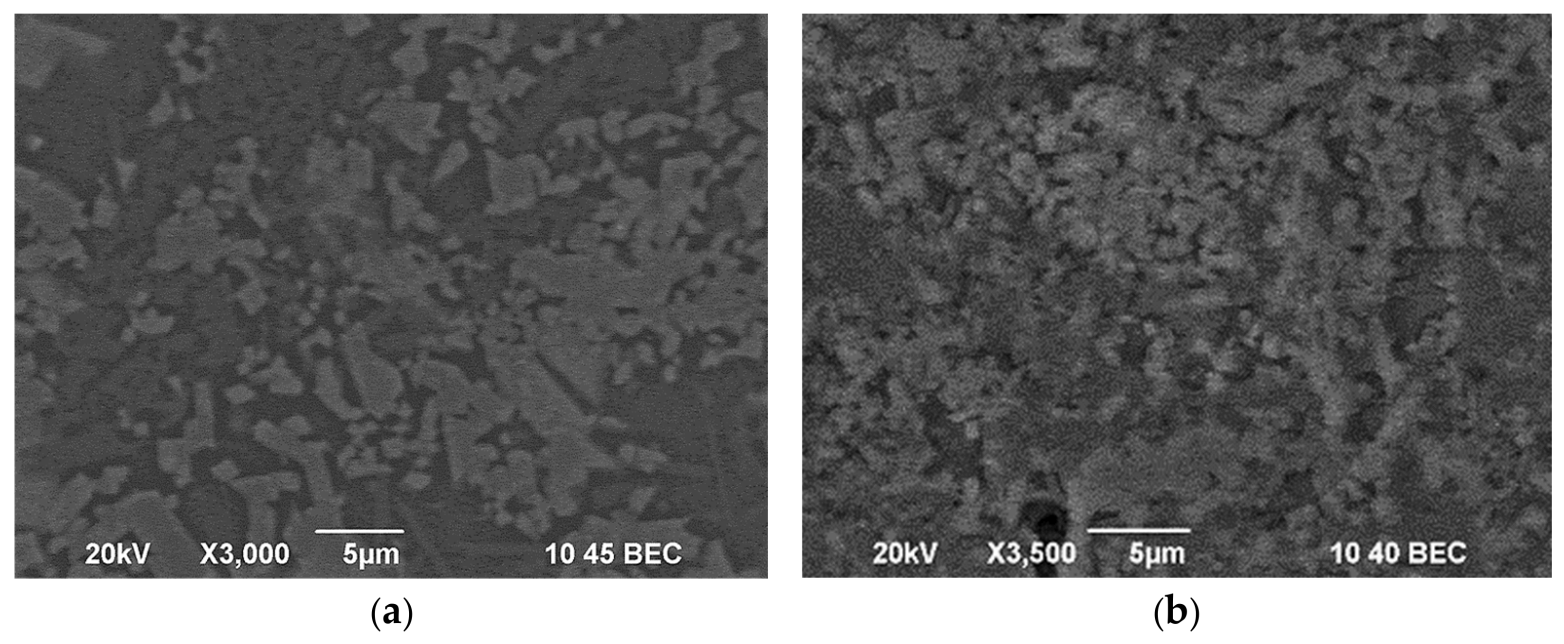
Original “Keramit” (**a**) and new ceramic C9/1 (**b**) at higher magnification.

Figure 8 shows the well-sintered structures of both ceramics and the formation of closed pores with spherical or near-spherical forms and sizes mostly below 10–20 microns. The high crystallinity and the formation of fine anorthite and pyroxene crystals with sizes mainly between 1 and 5 microns are demonstrated in Figure 9.

**Figure 10:**
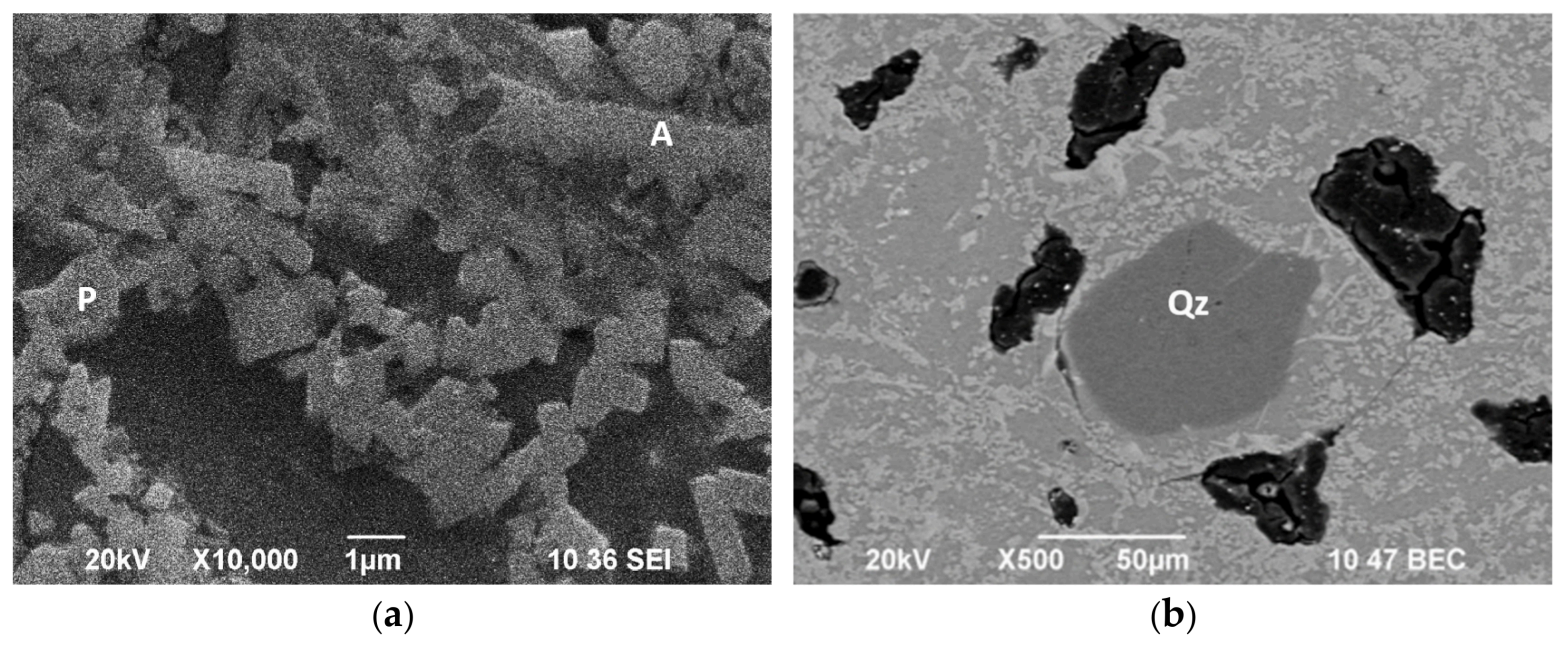
Anorthite (**A**) and pyroxene (**P**) crystals in ***C9/1*** (**a**) and residual quartz (**Qz**) in “Keramit” (**b**).

Figure 10a shows an image of sample ***C9/1*** at higher magnification; the EDS results for the main crystal phases are included in the Supplementary Materials: the EDS spectra of anorthite, pyroxene and quartz crystals are presented in Figures S1, S2 and S3, respectively, while the corresponding chemical analysis are summarized it Table S1. Together with CaO and MgO, the pyroxene crystals also contain some Al_2_O_3_ and Fe_2_O_3_ and practically no sodium oxide, which means that they can be described as solid solutions between Diopside (CaMgSi_2_O_6_), Esseneite (CaFeAlSiO_6_) and Ca-Tschermak’s Molecule (CaAlAlSiO_6_). The plagioclase is close to anorthite, with minor inclusions of Na_2_O, K_2_O and F_2_O_3_. These results are comparable with those of the original material [33].

In both ceramics some residual quartz is also observed. An example of such an undissolved crystal, with a size of 50 microns and typical cracks formed due to the beta–alpha transition of the quartz [14,34], is demonstrated in Figure 10b.

This fine crystalline structure can be explained by the specific nature of phase formation in the marl ceramics [35,36]. In practice, this is an interesting example of the Hedvall effect, in which the increased atomic mobility during the decomposition of calcite and the breakdown of clay minerals promotes the intensive formation of crystal nuclei, leading to a fine crystalline structure [37].

It can be concluded that the combination of an excellent degree of densification, coupled with the high crystallinity of the tiny pyroxene and anorthite crystals, is the main reason for the excellent performance of this material.

## 4. Conclusions

It has been demonstrated that by adding asbestos waste with high MgO content to local Bulgarian marl, it is possible to synthesize a ceramic clinker that is very similar to the original “yellow cobblestones” that cover the historic center of Sofia and are considered part of its cultural heritage.

The asbestos waste is mixed with the main part of the marl, the mixture is wet-milled and heat-treated at 1000 °C for thermal inertization of the asbestos and preliminary calcination. The resulting grog is then milled again, mixed with the remaining part of the marl and pressed. The samples produced in this way are heat-treated at 1160 °C for 4 h.

The chemical and phase compositions of the resulting ceramic, as well as its structure, are comparable to those of the original historical clinker. Both materials are characterized by a high crystallinity and the formation of tiny crystals of pyroxenes and anorthite, combined with an excellent degree of sintering and near-zero water absorption. This rare combination explains the very good properties of this outstanding paving ceramic, which has withstood more than a century in the heart of Bulgaria’s capital.

## Figures and Tables

**Table 1:** Chemical compositions (wt %) of “Keramit”—***K***, marl—***M***, asbestos waste—***A*** and final ceramic—***C9/1***.

	*K*	*M*	*A*	*C9/1*
SiO_2_	53.5	50.0	47.9	49.9
TiO_2_	0.6	0.7	-	0.6
Al_2_O_3_	14.6	16.7	2.8	15.5
Fe_2_O_3_	5.2	5.1	1.8	5.4
CaO	17.1	19.7	0.2	19.1
MgO	5.1	2.8	46.8	5.7
K_2_O	2.7	2.9	0.3	2.7
Na_2_O	1.1	1.5	0.1	0.9
SO_3_	0.1	0.5		0.2

**Table 2:** Temperatures and viscosities corresponding to the “fixed” points for G*9/1 and ***M***.

	log (η)	*M*	G*9/1
First Shrinkage (***T_FS_***)	9.0	1172	1145
Maximum Shrinkage (***T_MS_***)	7.2		1208
Softening (***T_S_***)	5.1	1186	1214
Half-Sphere (***T_HS_***)	3.6	1194	1225
Flow (***T_F_***)	3.1	1199	1231

**Table 3:** Densities and porosities of “Keramit”—***K*** and final ceramic—***C9/1***.

	*K*	*C9/1*
Apparent density (g/cm^3^)	2.54 ± 0.01	2.58 ± 0.01
Skeleton density (g/cm^3^)	2.564 ± 0.002	2.592 ± 0.002
Absolute density (g/cm^3^)	2.851 ± 0.002	2.903 ± 0.002
Water soaking (%)	0.4 ± 0.1	0.2 ± 0.1
Open porosity (%)	1.0 ± 0.2	0.5 ± 0.2
Closed porosity (%)	10.1 ± 0.2	10.7 ± 0.2

## Data Availability

The original contributions presented in this study are included in the article/Supplementary Material. Further inquiries can be directed to the corresponding author.

## Supplementary Materials

The following supporting information can be downloaded at: https://www.mdpi.com/article/10.3390/ma19204330/s1, Figure S1: EDS spectrum of anorthite; Figure S2: EDS spectrum of pyroxene; Figure S3: EDS spectrum of quartz; Table S1: chemical compositions of anorthite, pyroxene and quartz.
