# Supplementary material for: Synthesis of “Yellow Cobblestones” of Sofia Using Asbestos Waste
Source: Materials (Basel). 2026 Oct 15;19(20):4330. doi: 10.3390/ma19204330 (PMC13707101; doi:10.3390/ma19204330)
Supplement: Supplementary file 1 [file materials-19-04330-s001.zip › materials-4602875-supplementary.pdf]

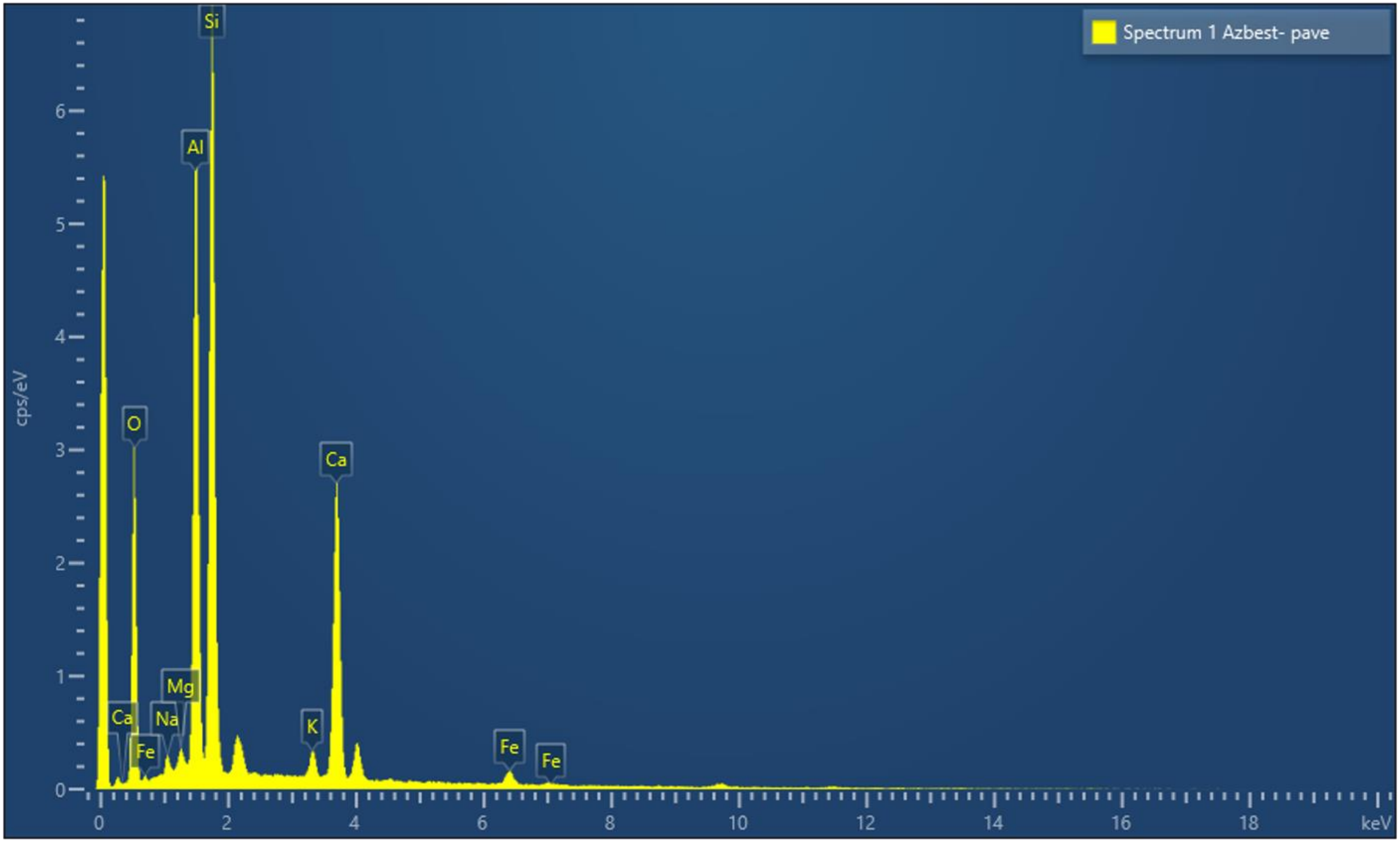

Figure S1: EDS spectrum of anorthite

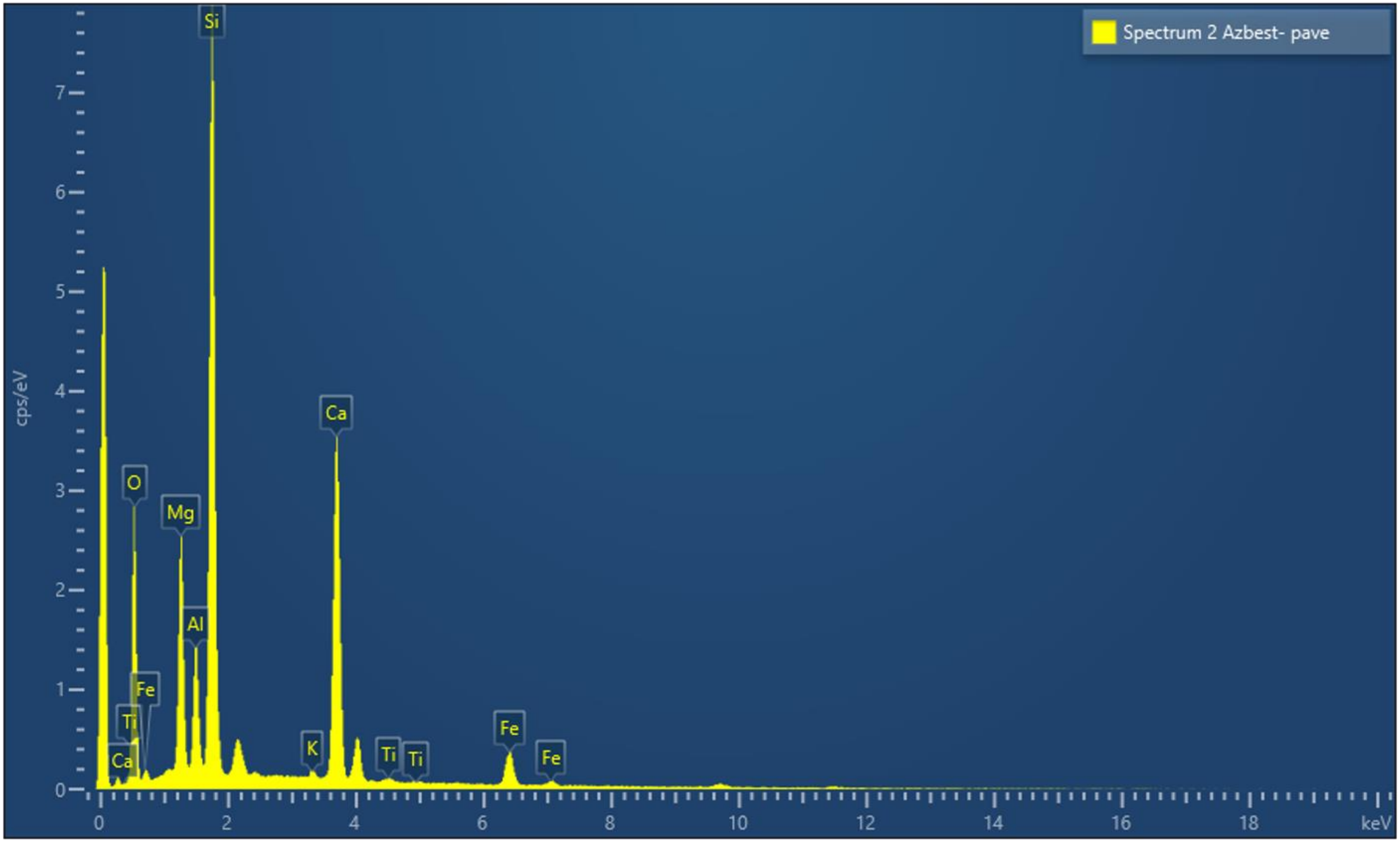

Figure S2: EDS spectrum of pyroxene

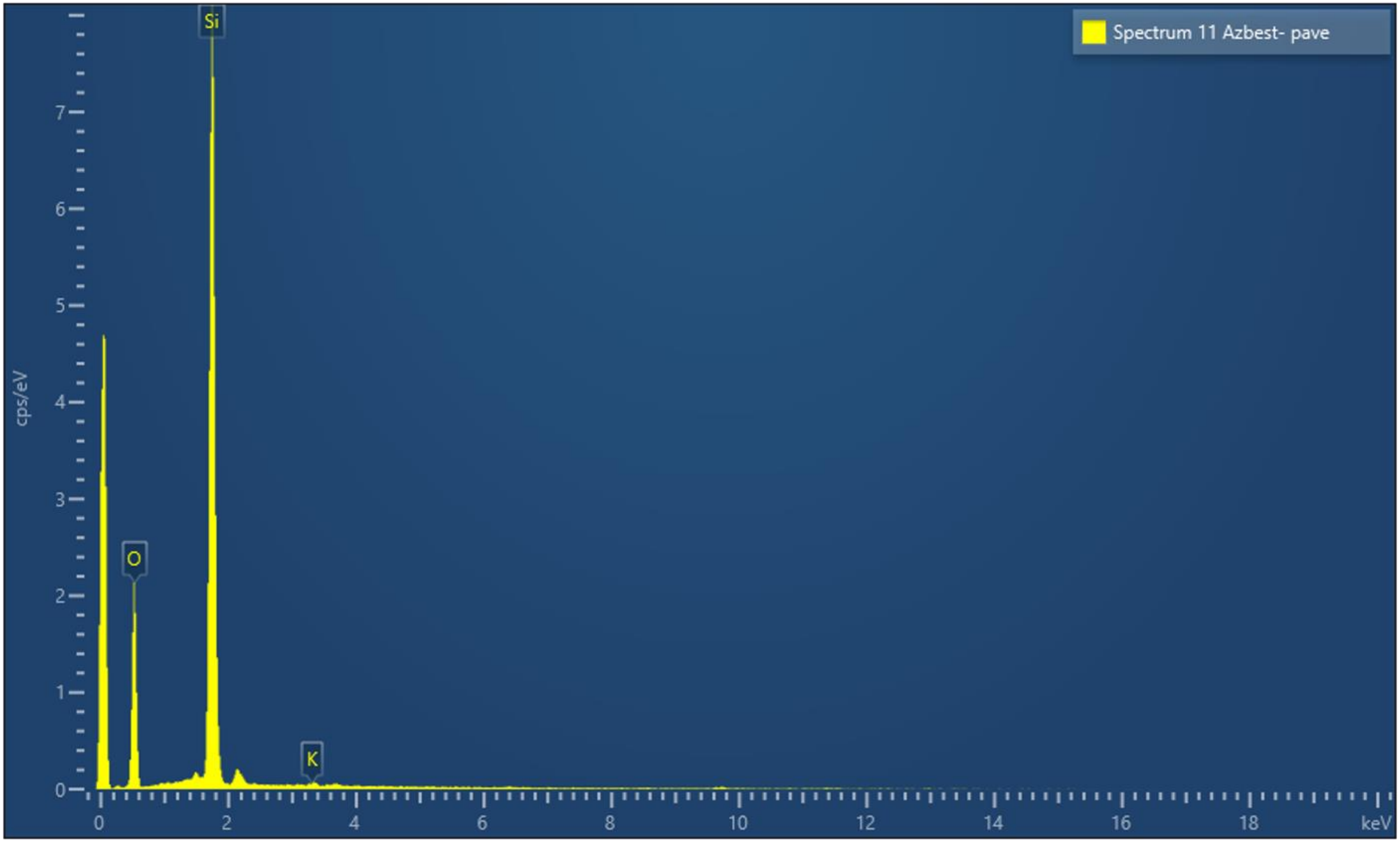

Figure S3: EDS spectrum of quartz.

Table S1: chemical compositions of anorthite, pyroxene and quartz.

	Spectrum 1 - anorthite	Spectrum 2 - pyroxene	Spectrum 11 – quartz
Na_2O	1.03		
MgO	0.91	13.16	
Al_2O_3	29.58	7.65	
SiO_2	46.71	48.85	99.58
K_2O	1.30	0.41	0.42
CaO	18.25	23.12	
TiO_2		0.40	
Fe_2O3	2.21	6.41	
Total	100.00	100.00	100.00
